# Spatial Directionality Found in Frontal-Parietal Attentional Networks

**DOI:** 10.1155/2018/7879895

**Published:** 2018-08-30

**Authors:** Gahangir Hossain, Mark H. Myers, Robert Kozma

**Affiliations:** ^1^Electrical Engineering and Computer Science, Texas A&M University-Kingsville, Kingsville, TX, USA; ^2^Anatomy and Neurobiology, University of Tennessee Health Science Center, Memphis, TN, USA; ^3^Department of Mathematics, University of Memphis, Memphis, TN, USA

## Abstract

Research in last few years on neurophysiology focused on several areas across the cortex during cognitive processing to determine the dominant direction of electrical activity. However, information about the frequency and direction of episodic synchronization related to higher cognitive functions remain unclear. Our aim was to determine whether neural oscillations carry perceptual information as spatial patterns across the cortex, which could be found in the scalp EEG of human subjects while being engaged in visual sensory stimulation. Magnitude squared coherence of neural activity during task states that “finger movement with Eyes Open (EO) or Eyes Wandering (EW)” among all electrode combinations has the smallest standard deviation and variations. Additionally, the highest coherence among the electrode pairs occurred between alpha (8-12 Hz) and beta (12-16 Hz) ranges. Our results indicate that alpha rhythms seem to be regulated during activities when an individual is focused on a given task. Beta activity, which has also been implicated in cognitive processing to neural oscillations, is seen in our work as a manner to integrate external stimuli to higher cognitive activation. We have found spatial network organization which served to classify the EEG epochs in time with respect to the stimuli class. Our findings suggest that cortical neural signaling utilizes alpha-beta phase coupling during cognitive processing states, where beta activity has been implicated in shifting cognitive states.* Significance.* Our approach has found frontoparietal attentional mechanisms in shifting brain states which could provide new insights into understanding the global cerebral dynamics of intentional activity and reflect how the brain allocates resources during tasking and cognitive processing states.

## 1. Introduction

Electroneurological studies have focused on the loosely coupled neural networks that dynamically incorporate and bind several areas across the cortex during cognitive processing [1]. Synchronization between cortical neighborhoods has been identified as areas involved in perceptual activity. Recent studies report transient synchronization between parietal and frontal cortices where low frequency oscillations (7–14 Hz) have been proposed to coordinate activity between disperse cortical areas during visual processing [2]. Simultaneously, while beta oscillatory activity (13–30 Hz) seems to be slightly reduced in parietal neural sources, a strong and long-lasting enhancement of beta is present within frontal-parietal areas. Cortical processing after visual stimulus entails the participation of multiple and widespread brain areas, such as the parietal cortex [3] and frontal cortices [4, 5].

When an individual is engaged in a current task that demands a degree of attentiveness, the frontal and posterior parietal cortical areas of the brain have been implicated using various neuroimaging techniques. These cortical areas are referred to collectively as the frontoparietal attentional control. Cortical networking has been analyzed during these states in order to better understand how higher orders of cognitive processing after stimuli induced cortical communication between neural populations.

In a study featuring meditative activities, cortical networks were analyzed during the following states: mind wandering, or loss of focus, awareness of mind wandering, and attentional focus. The dorsolateral frontal cortex has been specifically implicated in active rehearsal, which consists of, “the repetitive selection of relevant representations or recurrent direction of attention to those items” [6]. The attentional focus phase consists of repetitive activities such as motor control movement. The lack of activation in parietal elements of the executive network during this phase may be related to the role of the parietal cortex in disengagement of attention rather than in focusing attention [7]. During the mind wandering phase, neural activity was detected in posterior cingulate cortex, medial frontal cortex, posterior parietal/temporal cortex, and parahippocampal gyrus. Therefore, a higher degree of neural activity is seen in the basal state of cortical activity versus the focused state, which involves repetitive activity.

It has long been known that cortical waveforms (coordinated oscillations among mesoscopic neural populations) vary their frequency with cognitive focus. Synchronized neural spiking activities are observed during focal attention because some stimulus representations must be enhanced at the expense of others. Consistent with a thalamic generator, low-frequency oscillations are stronger in the deeper layers of cortex that project to the thalamus [8]. However, previous work has shown a decrease in low-frequency synchrony within visual cortex during sustained attention [9], necessitating future work to better understand the role of these oscillations. During a search of a visual display, shifts of covert attention and its correlate primary visual area neurons synchronize to lower-frequency, beta (~25 Hz) oscillations across the frontal cortex. This suggests a lockstep between neural activity and periodic sampling of the external world via an attentional spotlight [10]. The network interactions for attention networks seem to originate in the frontal cortex, the brain region most associated with “executive” brain functions. Frontal cortical neurons during the attentive states reflect a shorter latency phase of signal than parietal area [11, 12].

When attention is focused, the visual cortex goes into rhythmic synchrony with a phase offset that suggests the frontal cortex is driving the parietal area [13]. Frontoparietal neural activity is consistent with facilitated stimulus processing of the repeated stimuli rather active recollection of the stimuli, or the creation of new memory representations for unfamiliar stimuli (Henson et al. 2007).

Thalamic connections to the cerebral cortex may be the governing force regarding modulated cortical activity. The thalamus is responsible for the relaying of sensory and motor signals to the cerebral cortex, and the regulation of higher order cognitive processing. According to Baars [14], direct brain recording suggests that task-specific activity involves cross-frequency coupling at multiple spatial scales, linking, and unlinking multiple sites in the cortical network and it satellites. Signaling between subcortical populations may involve gamma and alpha-range synchrony in the cognitive processing of ambiguous stimuli. Our work reinforces the idea that alpha and beta activity in large populations of cells has been observed in motor activities as well. Preliminary results of our interpersonal coordination studies using scalp EEG arrays have been reported in [15]. Alpha (10 Hz) oscillations may serve to group faster rhythms such as beta and gamma. Altogether, neural signaling utilizes alpha-beta phase coupling during cognitive processing states.

Neural information processing has been investigated in terms of the amplitude and phase modulations of neural oscillatory behavior [16]. As the cortex processes incoming stimulus, action potentials convey the stimuli into “information” pulse trains. These pulse trains transition from background cortical activity to a higher order state of cortical activity that is measured as phase transitions. Previous experiments on the rabbit model have shown that phase transitions occur in the neural signaling rates of the theta and alpha ranges [17–19]. Synchronized oscillatory activity between neural networks across the cortex has shown that the phase of the signal between the networks remains constant during this intermittent time period.

Palva & Palva [20] and Lobier et al. [21] discussed potential issues with volume conduction in EEG connectivity analyses that have critical implications for any interpretations of scalp data. Brunner & Makeig [22] performed both analytical and numerical simulation and showed that the Directed Transfer Function (DTF) is influenced by volume conduction, whereas Kaminski and Blinowska [23] introduce the popular connectivity measures derived from VAR models that include the DTF and argue that DTF is not affected by volume conduction. Study from Brunner & Makeig [22] also argues that source activities can be obtained by separating each data channel signal into a sum of physically and physiologically distinct source processes whose interrelationships can also be modeled in terms of causal connectivity. This study focuses on the cortical neural networks of human cohorts and examines the neural information transmission behavior after visual stimulation.

## 2. Methods

### 2.1. Data Collection Methodology

The experiments for this project were held in the Computational Neurodynamics Lab at the FedEx Institute of Technology at the University of Memphis. This study has been approved by the University of Memphis Institutional Review Board (IRB-071411-790). Ten participants (4 females, 6 males, mean age 29) undergo ten recordings on the experimental protocol listed in Table 1. All participants had normal or corrected-to-normal vision and had no known psychological or neurological deficits. Participants were paid ten dollars for taking part in the experiment.

**Figure 1:**
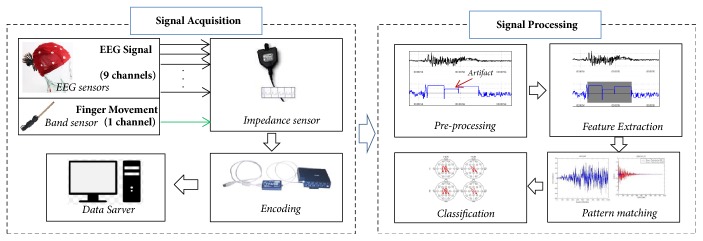
Event-related potentials (ERP) were captured via an EEG cap using nine electrodes. Additional sensors were utilized to capture finger movement via a bend sensor. Grounding and negative connections were facilitated via ear lobe connections. Positive, negative, and grounding connections were inserted into an impedance sensor, which connected to the Flex/Pro-comp Infinity™ amplifier. EEG data capture was accomplished through BioInfinity™ where data was exported in order to enable EEG analysis of cognitive states.

In the protocol we follow [24]. A participant is facing a monitor with an EEG cap and bend sensor wrapped around their finger. The monitor provided directions for the participant to perform as shown in Figure 1. Participants are sitting upright and receiving instructions from a monitor during the entire session, even during rest periods. During the “Eyes Wandering” activity, the participants were instructed to focus their eyes away from the monitor. Data was collected from BioInfinity's system for EEG cortical measurements and EMG movement via a bend sensor around the individual's finger. An EEG amplifier was provided using the Flax/Pro-comp InfinityTM amplifiers with the standard 10-20 electrode cap using 19 Ag/AgCl electrodes. The sampling rate was 2048 Hz.

### 2.2. Locating Phase Patterns

Table 2 illustrates the methodology to determine phase directionality. After the recordings are captured, magnitude squared coherence is initially performed on each reference to working electrode combination (F3-Fz, F3-F4, F3-C3…, Fz-F3, Fz-F4, Fz-C3… etc.) from 1-40 Hz, for dominant delta, theta, alpha, beta, and gamma frequencies. For each state of the protocol, magnitude squared coherence is averaged on the multiple recordings for each participant, and standard deviation and variation are calculated from the data collected. For each frequency bandwidth, electrode pairs with a coherence > 0.6 will be considered significant. The calculation of coherence is as follows.

Consider two signals x(t) and y(t), which are transformed to discrete sequences consisting of N uniformly spaced points *x*_*j*=_*x*(*t*_*j*_) and *y*_*j*=_*y*(*t*_*j*_), where *N* = 2^*n*^ with an integer, and *t*_*j*_ = *j*∆*t* where* j = 0* to* N-1*. The cross-correlation function CCF of x(t) and y(t) is defined as follows:

(1) $$\begin{array}{c} {CCF}_{xy}\left(\;\tau \;\right)=E\left(\;y_{n+\tau }{x_{n}}^{*}\;\right) \end{array}$$

where −*∞* < *n* < *∞*, E(·) is the expected value of the random variable, and x*∗* stands for complex conjugate. Next we use the Fast Fourier Transform (FFT), which breaks down a signal into constituent sinusoids of different frequencies. FFT is useful in signal processing for converting data from the time domain into the frequency domain. FFT is calculated using Discrete Fourier Transform (DFT) as follows:

(2) $$\begin{array}{c} X\left(\;\omega \;\right)=\overset{N-1}{\underset{j=0}{\sum }}x_{j}\exp\left(\;-\omega 2\mathrm{\pi i}\frac{j}{N}t\;\right) \end{array}$$

where *X*(*ω*) is the Fourier representation in frequency domain. EEG frequency data is typically found using power autospectral density function *PSD*_*X*_(*ω*), defined as |*X*(*ω*)|^2^. The power spectral density *CPSD*_*XY*_(*ω*) is defined as the Fourier Transform of *CCF*_*xy*_(*τ*) defined in (1). The squared coherence is given as follows:

(3) $$\begin{array}{c} \gamma^{2}=\frac{{CPSD}_{XY}\left(\omega \right)}{{PSD}_{X}\left(\omega \right){PSD}_{Y}\left(\omega \right)} \end{array}$$

Given the real and imaginary parts of each electrode signal from the FFT, we unwrap the phase of the signal in order to return the phase angle in radians. For all the channels, the mean and the standard deviation were calculated. Slope of the mean values is plotted in reference to each channel.

## 3. Results

**Figure 2:**
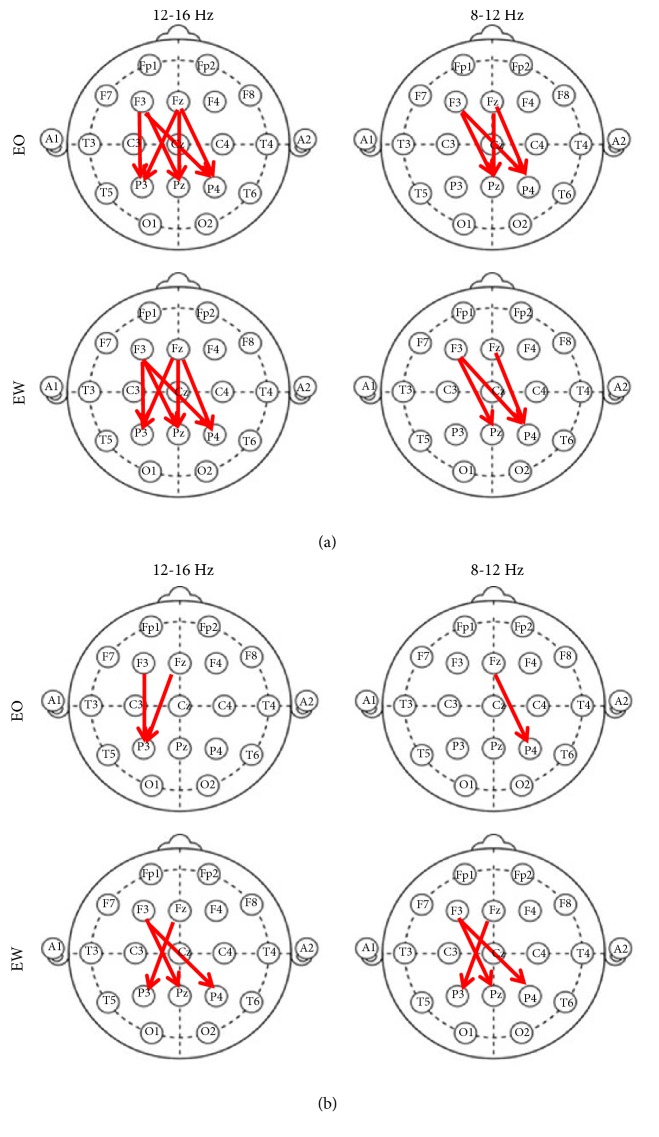
Magnitude squared coherence ((a) red arrows) has high coherence values (> 0.6) for the pairs marked by red arrows for the activity states, “Eyes Open + finger movement (EO)”, and “Eyes Wandering + finger movement (EW)”. Among all electrode combinations, the electrode pairs displayed above have the highest coherence values, while having small standard deviation. The highest coherence among the electrode pairs occurred between 8 and 12 Hz (alpha) and 12 and 16 Hz (beta) ranges. (b) Red arrows represent phase leads of the calculated mean of the unwrapped phases between channels.

**Figure 3:**
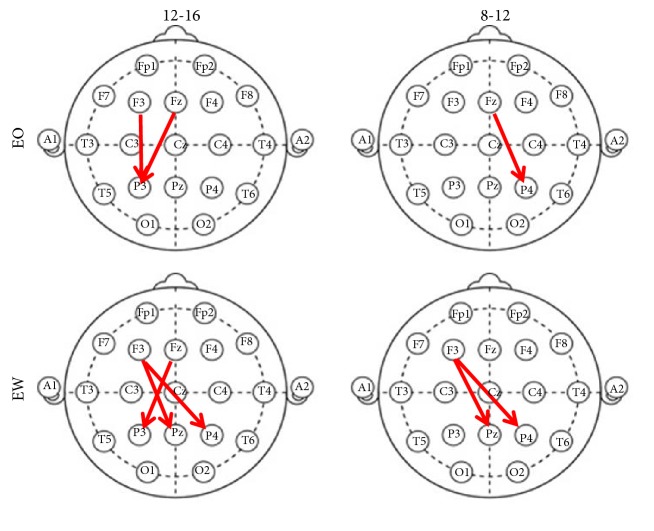
Overlapping directionality shared between magnitudes squared coherence values and unwrapped phase differences between channels.

The calculations that produce the smallest standard deviation and variation will have demonstrated which set of signal pairs have similar cross-spectral density across multiple recordings. Of all the signal pairings among frontal and parietal electrodes, F3 and Fz to P3, Pz, and P4 had the least amount of deviation between multiple recordings. Our results show that the highest coherence (> 0.6) among the electrode pairs occurred between 8 and 12 Hz (alpha) and 12 and 16 Hz (beta) ranges.  Figure 2(a) displays electrode pairs with high coherence values (red arrows) during states 2 and 4, i.e., Eyes Wandering + finger movement and Eyes Open + finger movement, respectively. Synchronous neural activity as it pertains to cognitive task activity is found in the unwrapped phase differences in Figure 2(b). The unwrapped phases in Figure 2(b) correspond to the results in Figure 3 as a subset of coherence in Figure 2(a) found between reference and working channels.

As the individual moves the bend sensor, their EEG signal transitions from nonlinear neural activity to synchronous activity. Of all the signal pairings among frontal, central, and parietal areas, reference electrodes F3 and Fz to neighboring electrodes P3, Pz, and P4 had the highest coherence between multiple recordings.

Overlapping directionality between magnitudes squared coherence values and unwrapped phase differences between channels are shown in Figure 3. The states, “Eyes Open” and “Eyes Wandering”, demonstrated low standard deviation and variance between recordings and low coherence between electrode pairs.

**Figure 4:**
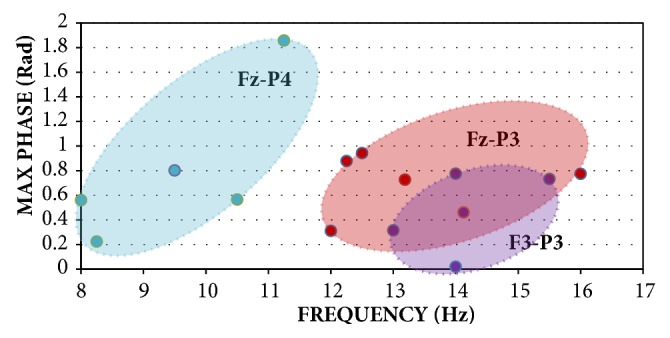
Cluster distributions of max-unwrapped phase values across 8-12 Hz and 12-16 Hz with respect to reference electrodes F3 and Fz for the “Eyes Open” state. Larger icons represent average of max values within a cluster, with standard deviation bars.

**Figure 5:**
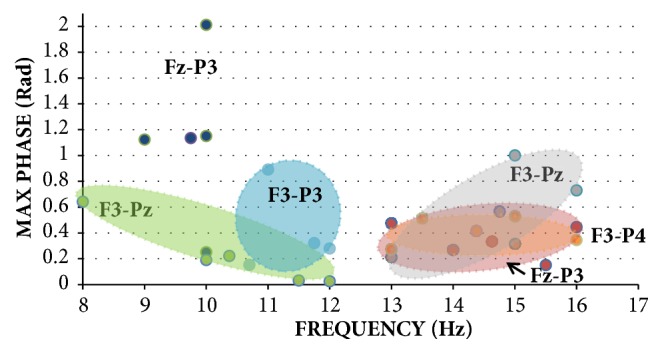
Cluster distributions of max-unwrapped phase values across 8-12 Hz and 12-16 Hz with respect to reference electrodes F3 and Fz for the “Eyes Wandering” state. Larger icons represent average of max values within a cluster, with standard deviation bars.

The distribution of the unwrapped phase values displays the similarity between EEG recordings per activity state. In Figure 4, during the “Eyes Open” state, the “Fz-P3” group has the smallest standard deviation grouping compared to the other clusters, followed by “F3-P3” and finally “Fz-P4”. In Figure 5, during the “Eyes Wandering” state, the “Fz-P3” and “F3-P4” group has the smallest standard deviation grouping compared to the other clusters, followed by “F3-Pz” for 8-12 Hz and 12-16 Hz, and finally “Fz-P3”. For each electrode pair, the slope was calculated to demonstrate the closeness of the distribution of each recording per state, seen in Table 3. Slope fitting was accomplished on those electrode groupings with the lowest standard deviation and variation as seen in Figures 4 and 5. The size of the distribution demonstrates the similarity of each neural response within a given state. The tight distribution between electrode pairs also demonstrates the similarity of the phase of the signal between recordings.

## 4. Discussion and Conclusion

In this work we studied spatial directionality of neural signatures of cognitive processing during task engagement and rest states using a standard 10-20 EEG system. The experiments involved a protocol with Eyes Open, Eyes Wandering, and Eyes Closed states of 10 subjects. Our results indicate that the calculated phase and coherence of EEG signals provide quantitative measures to determine the dominant interacting regions and the direction of electrical activity in the brain. Phase directionality is less pronounced during the resting state of the cortex. However, phase directionality of the electrical activity of the cortex appears to align in a dominant direction during cognitive activities, specifically during Eyes Open and Eyes Wandering + Finger Movement states. During these states, neural activity is diminished except for frontoparietal networks. These finding reflects how the brain allocates its resources during tasks that demand an individual's attention. Our main findings are summarized as follows:

- In our work, visual stimulus was used to engage areas of the brain in order to measure cortical alignment through task engagement. Our results indicate that alpha rhythms seem to be regulated during activities when an individual is focused on a given task. Beta activity, which has also been implicated in cognitive processing to neural oscillations, is seen in our work as a manner to integrate external stimuli to higher cognitive activation. Our results are in line with work of Dumenko [25], as well as [26], in which subjects were exposed to either a single stimulus in order to familiarize them to that stimulus or two stimuli in order to perform a discrimination task. The experiment [24] was designed to investigate perceptual or cognitive differences that might emerge when subjects experienced task engagement.
- Our additional remarkable result is that all electrodes contributed to the spatial network organization, which served to classify the EEG epochs in time with respect to the stimuli class, regardless of amplitude or variance. These findings are supported among forty recordings between the participants where frontoparietal networks exhibited the lowest standard deviation and highest degree of synchrony. Our finding is consistent with the evidence from widespread intermittent synchronization of ECoG patterns in rabbits and cats (Freeman and Burke 2003; Freeman and Rogers 2003) and intermittent synchronization of EEG patterns from a 1D array extending over 189 mm of the scalp [17, 27]. The primary method used is the Fast Fourier Transform to calculate the phase for spatial alignment found during cognitive tasking.
- Dominant neural network activity found during shifting cognitive states, specifically the averages and standard deviation of the dominant F3-P3 and Fz-P3 coherence, and phase directionality shown in Figure 3. Our results support Baars' [14] Global Workspace Dynamics, where task-specific activity involves cross-frequency coupling at multiple spatial scales, linking, and unlinking multiple sites in the cortical network and it satellites. Our findings suggest cortical neural signaling utilizes alpha-beta phase coupling during cognitive processing states, where beta activity has been implicated in shifting cognitive states [17, 18, 27, 28].

Our results support recent findings on frontoparietal attentional control networks. Specifically, Szczepanski and colleagues have found that frontoparietal networks are engaged in attentional allocation in both humans and nonhuman primates [29]. Their work supports Buschman's idea (2009) that while the participant was engaged in a visual stimulus directing them to move their finger, a shifting of attention and its correlate primary visual area neurons synchronize to lower-frequency, beta oscillations across the frontal cortex, suggesting a lockstep between neural activity and periodic sampling of the external world via an attentional cognitive states. Additional work has found that while a participant is focusing on a task, such as our “Eyes Open + Finger Movement” task, a cluster in the dorsolateral frontal region of the executive network remained active [30, 31]. This may represent persistent neural activity, i.e., keeping the goal in mind, to maintain sustained attention on the focal object [32, 33]. During the Eyes Wandering + Finger Movement state, Hasenkamp et al. [30] detected activity in posterior cingulate cortex, medial frontal cortex, posterior parietal/temporal cortex and parahippocampal gyrus, supporting our findings of frontoparietal activated networks. The frontoparietal executive network becomes active as participants disengage from focused attention to mind wandering where this pattern of shifting activity is consistent with an alternation between default mode and task-positive networks. The frontoparietal attentional network is observed in our study when we see two individuals engage their attention during the “Eyes Open + Finger Movement” task, where alpha activity is dominant. The individuals disengage their attention to the executive network where beta activity occurs during “Eyes Wandering + Finger Movement”.

The approach introduced in this paper provides new insights into understanding the global cerebral dynamics of intentional activity. Our analysis supports the idea that the sequence formation of frames begins with the abrupt resetting of phase values on every channel, followed by resynchronization and spatial pattern stabilization within the frame [16, 34]. Some results suggest [35] that scalp EMG can be attenuated by low-pass spatial filtering, using presently available arrays of 256 electrodes [36] and foreseeable arrays with exceptionally high density of recording and high sampling rates.

## Figures and Tables

**Table 1:** Experimental protocol.

**State**	**Time(s)**	**Activity**
1	1-40	Rest period
2	40-50	Eyes Open + finger movement
3	50-60	Rest period
4	60-70	Eyes Wandering + finger movement
5	70-80	Rest period
6	80-90	Eyes Closed + finger movement

**Table 2:** Signal processing procedure.

**Steps**	**Description**
(1) Data Preprocessing	Low-pass filter – Focus on frequencies below 40Hz.

(2) Signal Decomposition	Fast Fourier Transform – Extract real and imaginary parts of the signal.

(3) Electrode Pair Selection	Magnitude squared coherence is initially performed on electrode pairs considered to have significant coherence (> 0.6) for selection

(4) Unwrap Phase Function	The “unwrap” phase function is applied in order to return the phase angles in radians

(5) Mean and standard deviation calculation	For all the channels, the mean and the standard deviation were calculated.

(6) Directionality property analysis	Directionality is determined by the slope of mean of the unwrapped phases, which is either positive or negative, thereby constituting phase lead or phase lag. Phase directionality is designated as phase leads of the unwrapped phases between channels. Slope values are plotted to determine phase directionality in reference to each channel.

**Table 3:** Slope values of each recorded electrode pair, per state.

	F3-P3	F3-Pz	F3-Pz	F3-P4	F3-P4	Fz-P3	Fz-P3	Fz-P4	All
	(12-16 Hz)	(8-12 Hz)	(12-16 Hz)	(8-12 Hz)	(12-16 Hz)	(8-12 Hz)	(12-16 Hz)	(8-12 Hz)	
EW		m=0.017	m=0.040	m=0.036	m=0.029	m=0.116	m=0.022		m=0.038
		r^2^=0.262	r^2^=0.121	r^2^=0.003	r^2^=0.150	r^2^=0.005	r^2^=0.235		r^2^=0.158
EO	m=0.035					m=0.054		m=0.089	m=0.053
	r^2^=0.143					r^2^=0.138		r^2^=0.229	r^2^=0.006

## Data Availability

This experiment is conducted by us. No data is used from others.
